# The Outcomes of Total Anomalous Pulmonary Venous Connection in Neonates−10-Year Experience at a Single Center

**DOI:** 10.3389/fcvm.2021.775578

**Published:** 2021-11-12

**Authors:** Erchao Ji, Hailong Qiu, Xiaobing Liu, Wen Xie, Rong Liufu, Tao Liu, Jimei Chen, Shusheng Wen, Xiaohua Li, Jianzheng Cen, Jian Zhuang

**Affiliations:** ^1^Department of Cardiovascular Surgery, Guangdong Cardiovascular Institute, Guangdong Provincial People's Hospital, Guangdong Academy of Medical Sciences, Guangzhou, China; ^2^School of Medicine, South China University of Technology, Guangzhou, China; ^3^Department of Biostatistics School of Public Health, Brown University, Providence, RI, United States

**Keywords:** congenital heart disease, total anomalous pulmonary venous connection, pulmonary venous obstruction, neonate, survival, central venous pressure

## Abstract

**Background:** Recent developments in surgical techniques and hospital care have led to improved outcomes following repair of total anomalous pulmonary venous connection (TAPVC). However, surgical repair of neonatal TAPVC remains associated with a high risk of postoperative mortality and pulmonary venous obstruction (PVO). We conducted this retrospective study to identify risk factors associated with surgical outcomes in the neonatal population.

**Methods:** A retrospective review was conducted for all 127 neonates who underwent operations for isolated TAPVC from January 2009 to January 2019.

**Results:** Preoperative PVO occurred in 33 (26.0%) of the 127 patients. Fifty patients (39.4%) required tracheal intubation before the operation. Twenty-three patients (18.1%) underwent emergency surgery. There were 11 (8.7%) early deaths. Significant risk factors were prolonged cardiopulmonary bypass (CPB) time (*p* = 0.013) and increased postoperative central venous pressure (CVP, *p* = 0.036). There were 5 (4.3%) late deaths within 1 year of repair. The risk factors for overall death were preoperative acidosis (*p* = 0.001), prolonged CPB time (*p* < 0.001) and increased postoperative CVP (*p* = 0.007). In particular, mortality was significantly higher (*p* = 0.007) with a postoperative CVP > 8 mmHg. With an increase in use of sutureless techniques (*p* = 0.001) and decrease in deep hypothermic circulatory arrest (*p* = 0.009) over the past 5 years, postoperative mortality greatly decreased (21.2%: 6.7%, *p* = 0.016). Postoperative PVO occurred in 15 patients (11.8%). Risk factors were mixed TAPVC (*p* = 0.037), preoperative acidosis (*p* = 0.001) and prolonged CPB time (*p* = 0.006).

**Conclusion:** Although postoperative mortality of neonatal TAPVC has dropped to 6.7% over the past 5 years, it is still relatively high. Risk factors for postoperative death include preoperative acidosis, prolonged CPB time and increased postoperative CVP. Mortality was significantly higher for neonates with an average CVP > 8 mmHg 24 h after surgery.

## Introduction

Total anomalous pulmonary venous connection (TAPVC) is a rare but critical cardiac anomaly, accounting for approximately 2% of congenital heart disease cases (1). Its defining characteristic is that the pulmonary venous confluence does not flow into the left atrium, but rather the systemic venous system. Historically, TAPVC has been associated with a high mortality of 80% in the first year of life if there is no intervention (2), so it is often treated with surgery at an early stage. Since the first surgical attempt to correct TAPVC was reported in 1951 (3), improved surgical techniques and perioperative care have led to significantly better outcomes for TAPVC surgery in children (4, 5). However, compared with older children, the neonates are more vulnerable owing to less developed tissues and organs, which also leads to more difficult exposure and narrower surgical space. As reported from various research centers, newborns have a significantly higher risk of postoperative mortality and pulmonary vein obstruction (PVO), and are one of the TAPVC subgroups with the worst prognosis (6–8). This study was a retrospective analysis designed to identify risk factors associated with postoperative death and PVO in all neonatal patients who underwent TAPVC repair in our center in recent years.

## Materials and Methods

### Clinical Data

The Guangdong Provincial People's Hospital Institutional Research Ethics Board (2019338 H) approved the study. From January 2009 to January 2019, 127 consecutive patients who underwent TAPVC repair in the first month of their lives were identified in Guangdong Provincial People's Hospital. Clinical data were reviewed retrospectively. TAPVC patients with complex cardiac anomalies other than an atrial septal defect (ASD), patent ductus arteriosus, or ventricular septal defect were excluded. Echocardiography was performed on all patients. Computed tomography angiography (CTA) provided an alternative diagnostic modality for morphologic evaluation. It was used to help identify atypical vessels flowing into the systemic veins, show the courses and draining sites of the anomalously connected pulmonary vein (PV), and reveal obstructions in vertical veins (VV).

The patients were divided into two groups based on when their operation took place: January 2009 to December 2013 (Period 1) or January 2014 to January 2019 (Period 2). A diagnosis of PVO was made when echocardiographic data indicated a non-phasic flow velocity > 1.8 m/s. Emergency surgery was defined as a lifesaving operation performed ≤ 24 h after admission. Early mortality was defined as death occurring within 30 days of the operation or before hospital discharge. Preoperative and postoperative CVP were defined as mean central venous pressure within 24 h before and after surgery, respectively.

### Surgical Technique

#### Conventional Repair

All surgeries were performed under general anesthesia. A median sternal incision was routinely made, and cardiopulmonary bypass was established through regular intubation of the superior and inferior vena cava and ascending aorta. For supracardiac TAPVC, the right atrium was dissected, the common pulmonary and vertical veins were dissociated, the left atrium posterior wall and the common pulmonary vein anterior wall were dissected, and the left atrium and common vein were anastomosed. Then the ASD or foramen ovale were repaired through the right atrial incision. For cardiac TAPVC, the original atrial septal tissue was cut off, and the ASD was enlarged. The septal tissue between the coronary sinus (CS) and the left atrium was cut off to the posterior wall of the left atrium. The ASD was repaired and the pulmonary veins and the CS opening were separated to the left atrium. The left atrial appendage, common vein, and vertical vein were exposed in the infracardiac TAPVC through the oblique pericardial sinus. The distal end of the vertical vein was sutured and cut off. The common vein was cut along the proximal end of the fracture, and the posterior wall of the left atrium was cut and sutured. For the mixed TAPVC, the common pulmonary veins were anastomosed to the left atrium as described above, and the other individual returning pulmonary veins were separately anastomosed to the left atrium.

#### Sutureless Repair

Cardiopulmonary bypass was established using standard methods. The heart was turned into the right thoracic cavity, the posterior wall of the left atrium and the anterior wall of the common pulmonary vein were cut open longitudinally to four pulmonary vein openings, and the posterior wall of the left atrium was anastomosed with pericardium of the margin of the common venous cavity. Prior to 2014, sutureless technology was used sparingly in our center, mainly in patients with blood flow instability such as preoperative PVO. After 2014, with maturation of sutureless technology, we adopted sutureless technology for patients with supracardiac and infracardiac TAPVC. We generally do not perform an incision for a branched pulmonary vein unless it is severely obstructed.

### Statistical Analysis

All data were analyzed with IBM SPSS v25.0 (IBM, Armonk, NY) and STATA v16.0, software (StataCorp, College Station, TX). Normally-distributed continuous variables are reported as mean ± SD. Student's t tests were used to compare differences between groups. For skewed continuous variables, median (interquartile range [IQR]) was used to describe distributions, and the Wilcoxon-Mann-Whitney *U*-test was used to compare differences between groups. Descriptive statistics for categorical variables were reported as frequency/percentage and compared using the Pearson χ^2^ or Fisher exact test. Kaplan-Meier analysis was used to analyze rates of overall survival and freedom from postoperative PVO. Potential risk factors for binary outcomes (early mortality and overall mortality) were identified by logistic regression analysis. Cox proportional hazards analysis was used to identify hazards for time-related outcomes (postoperative PVO and reoperation). In multivariate analysis, *p* < 0.05 was used for variable inclusion and *p* > 0.1 for exclusion, and the forward stepwise method was used to remove the variables until the *p*-value of the remaining variable in the multivariate model was < 0.05. All tests were two-tailed, and a *p*-value < 0.05 was considered statistically significant.

## Results

### Baseline Characteristics

The analysis included 127 patients who underwent TAPVC repair at our institution during the study interval. The types of TAPVC were supracardiac in 53 patients (41.7%), cardiac in 24 (18.9%), infracardiac in 42 (33.1%), and mixed in 8 (6.3%). The median surgical age and weight were 13 days (IQR: 9–19 days) and 3.18 kg (IQR: 2.90–3.46 kg), respectively. In our study, 33 patients (26%) had preoperative PVO. Among them, 16 patients had vertical vein stenosis, 6 had stenosis at the common junction of pulmonary veins, 3 had stenosis caused by a restrictive atrial septal defect, 3 had stenosis where the vertical vein passed through diaphragm, 4 had stenosis caused by compression of the pulmonary veins by the aorta or pulmonary artery, and 1 had common pulmonary vein stenosis. The branched pulmonary veins were well-developed in these patients. Ventilation by tracheal intubation before the operation was used for 50 patients (39.4%), 15 (11.8%) had preoperative acidosis, and 25 (19.7%) underwent preoperative rescue. Due to hemodynamic instability and critical condition of patients, 23 (18.1%) underwent emergency surgery without examination by CTA in order to avoid potential risks during the transfer process and sedation during the examination (Table 1).

**Table 1:** Preoperative characteristics of 127 patients.

**Variable**	**No. (%) or mean ± SD or median (IQ range)**
**Demographics**	
Male: female	91:36
Age at surgery in days (IQ range)	13.0 (9-19)
Weight at surgery (kg)	3.17 ± 0.5
Length at surgery (cm)	50.0 (49-52)
**Diagnosis**	
Prematurity	9 (7.1)
Preoperative acidosis	15 (11.8)
Preoperative obstruction	33 (26.0)
Preoperative SPO_2_	86.0 (78.8-93.0)
Preoperative CVP	6.0 (5.0-8.0)
ASD	127 (100)
PDA	59 (46.5)
Preoperative PAH	121 (95.3)
**Anatomical type**	
Supracardiac	53 (41.7)
Cardiac	24 (18.9)
Infracardiac	42 (33.1)
Mixed	8 (6.3)
**Interventions**	
Preoperative intubation	50 (39.4)
Preoperative rescue	25 (19.7)
Emergency	23 (18.1)

*SPO2, oxygen saturation; CVP, central venous pressure; ASD, atrial septal defect; PDA, patent ductus arteriosus; PAH, pulmonary arterial hypertension*.

### Initial Surgical Procedures

Surgery was performed with standard CPB. Deep hypothermic circulatory arrest (DHCA) was required in 25 patients (19.7%), with significantly greater frequency in Period 1 than Period 2 [30.8% (16/52) vs. 12.0% (9/75), *p* = 0.009]. Ninety-three patients underwent a conventional operation and 34 patients were repaired by the sutureless technique. Vertical vein ligation was performed on 49 patients. Median CPB and aortic cross clamping time were 96 min (IQR: 76–118 min) and 49 min (IQR: 36–58 min), respectively. Delayed chest closure was required in 66 patients due to postoperative hemodynamic instability (median: 2 days; range: 1–21 days). Eight patients were intubated after operation. Three patients underwent re-intubation due to poor postoperative wound healing, 2 due to postoperative diaphragmatic paralysis, and 3 due to postoperative cardiopulmonary insufficiency. Seven patients were reoperated because of postoperative PVO. Postoperative median ventilation time was 110 min (IQR: 74–147) and the median stay in the cardiac intensive care unit (CICU) was 3 days (IQR: 2–4 days). Coagulation parameters, liver function and ECG were routinely monitored.

### Mortality

Sixteen patients (12.6%) died during the study period. A variety of ailments resulted in early mortality in 11 patients (8.7%). Low cardiac output due to heart failure was the cause of death for 5 of the 11 patients. Two died due to a pulmonary hypertensive crisis. One patient was in critical condition upon arrival at the hospital and underwent emergency operation, but died of intracranial hemorrhage. One died from obstruction of the superior vena cava. One developed coagulation dysfunction after an operation and died of multiple organ dysfunction syndromes. One died from severe renal failure. Later deaths in an additional five patients (3.9%) were due to postoperative anastomotic and branch pulmonary vein obstruction. Four of those patients died from reoperation that failed to relieve the obstruction; the fifth died of cardiogenic shock without reoperation.

**Figure 1:**
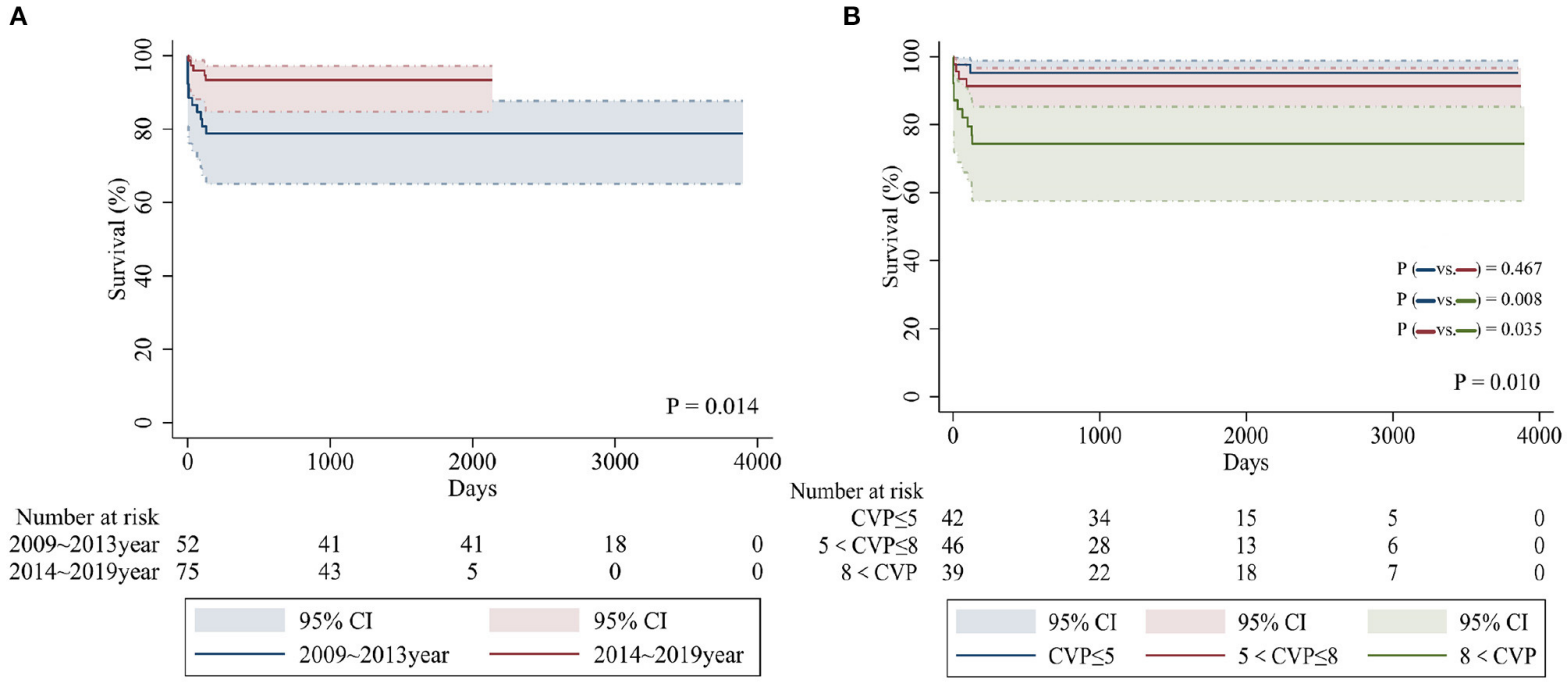
Kaplan-Meier curves comparing overall survival in patients from different time periods **(A)** and postoperative CVP values (mmHg) **(B)**.

Table 2 shows a breakdown of the results during the two consecutive periods (January 2009 to December 2013 and January 2014 to January 2019). There were significant differences in early and total mortality between the two periods (*p* = 0.05, *p* = 0.016). A comparison of survival for the two periods is shown in Figure 1A. There were no significant differences in the incidence of preoperative PVO between the two periods (*p* = 0.833). Compared with the first period, the incidence of preoperative acidosis and emergency surgery was reduced in the second period, but the difference was not statistically significant (*p* = 0.110, *p* = 0.093). When the second period was compared with the first, there were significantly more sutureless surgery patients (*p* < 0.001) and fewer patients who required intraoperative deep hypothermic circulatory arrest (*p* = 0.009). There were also significantly fewer cases of postoperative CVP increased in the second period (*p* = 0.007).

**Table 2:** Mortality and patient characteristics according to surgical period.

**Variable**	**Group 1 (2009-2013)**					**Group 2 (2014-2019)**					***P*-value**
	**Supracardiac**	**Cardiac**	**Infracardiac**	**Mixed**	**Overall**	**Supracardiac**	**Cardiac**	**Infracardiac**	**Mixed**	**Overall**	
Patients, *n*	24	9	15	4	52	29	15	27	4	75	0.722
Pre-acidosis	3 (12.5%)	1 (11.1%)	3 (20.0%)	2 (50.0%)	9 (17.3%)	2 (6.9%)	0	3 (11.1%)	1 (25.0%)	6 (8.0%)	0.11
Pre-PVO	9 (37.5%)	0	4 (26.7%)	0	13 (25.0%)	13 (44.8%)	0	7 (25.9%)	0	20 (26.7%)	0.833
Emergency	5 (20.8%)	2 (22.2%)	5 (33.3%)	1 (25.0%)	13 (25.0%)	5 (17.2%)	1 (6.7%)	4 (14.8%)	0	10 (13.3%)	0.093
Sutureless	3 (12.5%)	0	1 (6.7%)	0	4 (7.7%)	11 (37.9%)	1 (6.7%)	18 (66.7%)	0	30 (40.0%)	<0.001
DHCA	5 (20.8%)	0	10 (66.7%)	1 (25.0%)	16 (30.8%)	5 (17.2%)	0	4 (14.8%)	0	9 (12.0%)	0.009
Median post-CVP	8.8	5	8	6.8	8	7	7	6	5.5	7	0.007
Post-PVO	3 (12.5%)	1 (11.1%)	1 (6.7%)	3 (75.0%)	8 (15.4%)	3 (10.3%)	0	4 (14.8%)	0	7 (9.3%)	0.299
Reoperation	2 (8.3%)	1 (11.1%)	1 (6.7%)	1 (25.0%)	5 (9.6%)	1 (3.6%)	0	1 (3.7%)	0	2 (2.7%)	0.122
Early deaths	5 (20.8%)	0	3 (20.0%)	0	8 (15.4%)	2 (6.9%)	0	1 (3.7%)	0	3 (4.0%)	0.05
Mortality	5 (20.8%)	1 (11.1%)	3 (20.0%)	2 (50.0%)	11 (21.2%)	3 (10.3%)	0	2 (7.4%)	0	5 (6.7%)	0.016

*PVO, pulmonary venous obstruction; DHCA, deep hypothermic circulatory arrest; CVP, central venous pressure*.

Multivariable logistic regression analysis identified the following as independent risk factors for early death (Table 3): prolonged cardiopulmonary bypass time (odds ratio = 1.086, 95% confidence interval [CI]: 1.018–1.159, *p* = 0.013) and increasing postoperative central venous pressure (odds ratio = 2.092, 95% CI: 1.050–4.168, *p* = 0.036). Multivariate logistic regression analysis revealed the following risk factors for overall mortality (including late death) (Table 4): preoperative acidosis (odds ratio = 21.974, 95% CI: 3.425–140.965, *p* = 0.001), prolonged cardiopulmonary bypass time (odds ratio = 1.032, 95% CI: 1.015–1.049, *p* < 0.001), and increasing postoperative central venous pressure (odds ratio = 1.538, 95% CI: 1.127–2.100, *p* = 0.007) (Figure 1B).

**Table 3:** Univariate and multivariate analysis for early death.

**Variable**	**Univariable**			**Multivariable**		
	***P*-value**	**Odds ratio**	**95% CI**	***P*-value**	**Odds ratio**	**95% CI**
Emergency	0.021	4.537	1.250-16.463			
Acidosis	0.016	5.455	1.377-21.607			
Weight	0.015	0.199	0.054-0.731			
Age	0.041	0.895	0.805-0.995			
CPB	<0.001	1.051	1.027-1.076	0.013	1.086	1.018-1.159
Post-intubation time	0.001	1.006	1.003-1.010			
CICU	0.001	1.188	1.073-1.315			
Post-SPO_2_	<0.001	0.557	0.403-0.770			
Post-CVP	0.003	1.390	1.121-1.723	0.036	2.092	1.050-4.168
Post-PAH	0.013	2.216	1.175-3.847			
Surgical period	0.036	0.229	0.058-0.910			

*PAH, pulmonary arterial hypertension; SPO_2_, oxygen saturation; CVP, central venous pressure; CPB, cardiopulmonary bypass; CICU, cardiac care unit*.

**Table 4:** Univariate and multivariate analysis for overall death.

**Variable**	**Univariable**			**Multivariable**		
	***P*-value**	**Odds/hazard ratio**	**95% CI**	***P*-value**	**Odds/hazard ratio**	**95% CI**
Acidosis	<0.001	14.857	4.285-51.516	0.001	21.974	3.425-140.965
Weight	0.026	0.286	0.095-0.860			
Pre-SPO_2_	0.034	0.955	0.915-0.997			
Emergency	0.007	4.618	1.506-14.165			
CPB	<0.001	1.034	1.019-1.050	<0.001	1.032	1.015-1.049
Post-PAH	0.028	3.318	1.137-9.685			
Post-PVO	0.002	6.800	2.007-23.037			
Post-SPO_2_	<0.001	0.643	0.503-0.821			
Post-CVP	0.002	1.341	1.114-1.613	0.007	1.538	1.127-2.100
Post-intubation time	0.001	1.005	1.002-1.009			
CICU	0.001	1.174	1.064-1.295			
Reoperation	<0.001	24.773	4.291-143.005			
Surgical Period	0.021	0.266	0.086-0.820			

*PAH, pulmonary arterial hypertension; PVO, pulmonary venous obstruction; SPO_2_, oxygen saturation; CVP, central venous pressure; CPB, cardiopulmonary bypass; CICU, cardiac care unit*.

### Postoperative PVO and Reoperation

Among the 127 neonates with TAPVC, 33 (26.0%) had preoperative PVO with a tracheal intubation rate that was significantly higher than observed with neonates who did not (57.6 vs. 33.0%, respectively, *p* = 0.013). Postoperative PVO occurred in 15 patients (11.8%), 8 of whom suffered from anastomotic obstruction, 4 from anastomotic and branch pulmonary vein obstruction, and the other 3 from branch pulmonary vein obstruction. Seven patients (5.5%) underwent reoperation, after which 5 died. Eight patients had no indications for surgery at a subsequent follow-up, although 1 died from cardiogenic shock because of a delay in readmission. Pulmonary vein obstruction in the remaining seven patients was significantly alleviated at the subsequent follow-up.

**Figure 2:**
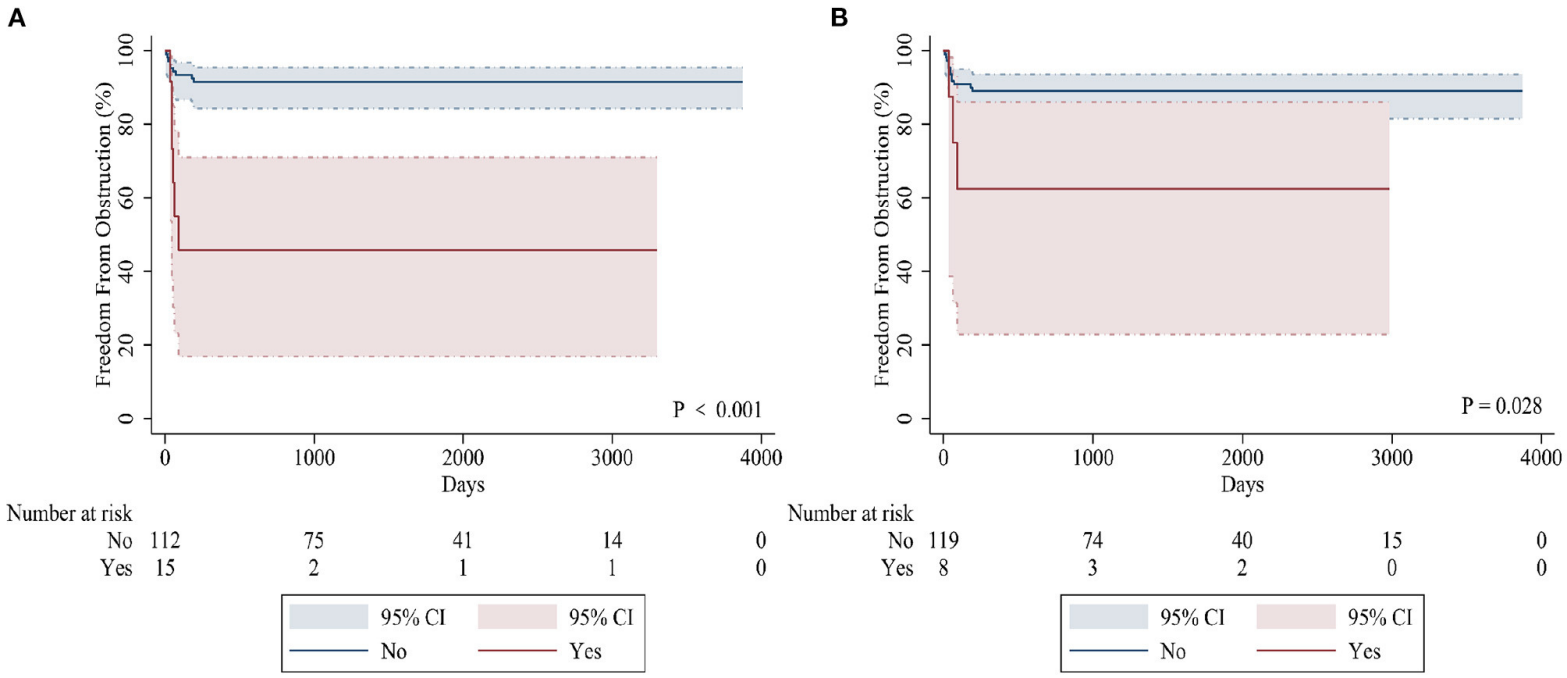
Kaplan-Meier curves comparing freedom from postoperative PVO in patients with preoperative acidosis **(A)** and mixed TAPVC **(B)**.

The sutureless technique was used for primary TAPVC correction in 34 patients. Although the incidence of PVO after operation was lower than for conventional surgery (8.8% [3/34] vs. 16.1% [15/93], *p* = 0.39), there was no statistically significant difference between them. The related risk factors of postoperative PVO were as follows: preoperative acidosis (hazard ratio = 5.791, 95% CI: 2.020–16.601, *p* = 0.001) (Figure 2A), prolonged cardiopulmonary bypass time (hazard ratio = 1.016, 95% CI: 1.005–1.028, *p* = 0.006) and mixed TAPVC (hazard ratio = 3.561, 95% CI: 1.326–12.967, *p* = 0.037) (Figure 2B). Preoperative acidosis and prolonged postoperative intubation time were independent risk factors for reoperation (Table 5).

**Table 5:** Univariate and multivariate analysis for overall reoperation and post-PVO features.

**Variable**	**Univariable**			**Multivariable**		
	***P*-value**	**Hazard ratio**	**95% CI**	***P*-value**	**Hazard ratio**	**95% CI**
**Post-PVO**						
Mixed	0.041	3.736	1.053-13.275	0.037	3.561	1.326-12.967
Acidosis	<0.001	7.691	2.763-23.557	0.001	5.791	2.020-16.601
Pre-SPO_2_	0.030	0.959	0.924-0.996			
CPB	0.002	1.016	1.006-1.027	0.006	1.016	1.005-1.028
Post-intubation time	0.002	1.003	1.001-1.004			
Post-PAH	0.005	2.002	1.227-3.266			
**Reoperation**						
Acidosis	<0.001	15.824	3.504-71.455	0.002	11.577	2.542-52.722
CPB	0.009	1.022	1.005-1.038			
Post-intubation time	0.002	1.004	1.002-1.007	0.003	1.004	1.002-1.007
Post-PAH	0.034	5.897	1.144-30.411			

*PAH, pulmonary arterial hypertension; PVO, pulmonary venous obstruction; SPO_2_, oxygen saturation; CPB, cardiopulmonary bypass*.

## Discussion

At present, the number of studies on the prognosis of neonatal TAPVC is relatively small. Neonates, especially those with preoperative obstruction, have a poor prognosis and mortality remains high (9). Therefore, we retrospectively reviewed all neonatal patients undergoing surgical correction of TAPVC in our center and analyzed the related risk factors for prognosis.

### Mortality

We found that when compared with previous studies of all age groups, the hospital and overall mortality rates of neonatal patients is significantly higher, consistent with previous research findings (9). Since 1995, hospital mortality of all age groups has been <5% (7, 10). However, neonates present the most difficult subgroup of patients with TAPVC. Their preoperative health is poor, often accompanied by hypoxia, acidosis, and dysplasia of organs, and postoperative mortality is high.

We divided the patients into two groups based on when their operations took place, and observed a decline in neonatal mortality between the first and second periods [21.2% (11/52) vs. 6.7% (5/75), respectively]. After further in-depth comparative analysis, we found that the following factors may have been associated with the decrease in mortality. First, the proportion of patients with preoperative acidosis or emergency operation decreased significantly. Preoperative acidosis increases pulmonary vascular reactivity and thus triggers postoperative pulmonary hypertensive events (11). In recent years, through further improvement of prenatal diagnosis, our center has formulated a postpartum perioperative management strategy. Implementation of a strategy of early diagnosis and treatment helps most children to be in a stable preoperative state and significantly reduces the incidence of preoperative acidosis. At the same time, as in previous studies (12, 13), we actively tried to use preoperative extracorporeal membrane oxygenation (ECMO) to stabilize the patient's condition prior to surgery, thereby reducing the need for salvage emergency operations. Second, the utilization rate of DHCA declined in the second experimental period compared to the first experimental period (12.0 vs. 30.8%). Prolonged DHCA may have some adverse effects on children's nervous and circulatory systems, and even cause multiple organ dysfunction. Most studies suggest that less use of DHCA reduces postoperative mortality (7, 14, 15). Our results are consistent with this conclusion. Third, since 2014, our center has routinely used sutureless surgery for the first correction of TAPVC. Our study found that sutureless technology improved surgical outcomes for patients with infracardiac TAPVC, preoperative PVO, low body weight, or complicated anatomy. These observations are also consistent with previous studies showing that sutureless technology is more suitable for high-risk patients with obstructive, infracardiac or mixed TAPVC (16–18). However, we cannot definitively conclude that sutureless technology is superior to conventional repair because the difference in overall mortality associated with the two approaches was not found to be statistically significant (Table 6).

**Table 6:** Comparison of two surgical methods (all patients and those admitted after 2014).

**Variable**	**Overall**			**2014-2019**		
	**Conventional**	**Sutureless**	***P*-value**	**Conventional**	**Sutureless**	***P*-value**
Infracardiac TAPVC	24.7% (23/93)	55.9% (19/34)	0.001	20.0% (9/45)	60.0% (18/30)	<0.001
Acidosis	10.8% (10/93)	14.7% (5/34)	0.544	2.2% (1/45)	16.7% (5/30)	0.035
Pre-PVO	21.5% (20/93)	38.2% (13/34)	0.057	20.0% (9/45)	36.7% (11/30)	0.110
Pre-intubation	36.6% (34/93)	47.1% (16/34)	0.284	33.3% (15/45)	53.3% (16/30)	0.085
Emergency	17.2% (16/93)	20.6% (7/34)	0.795	6.7% (3/45)	23.3% (7/30)	0.079
CPB	100.89	122.81	0.035	95.33	121.85	0.010
Post-ventilation time	132.62	188.89	0.113	111.01	154.88	0.061
CICU	3.5	5.2	0.064	3.3	5.3	0.054
Post-PVO	12.9% (12/93)	8.8% (3/34)	0.758	11.1% (5/45)	6.7% (2/30)	0.695
Early mortality	8.6% (8/93)	8.8% (3/34)	1	2.2% (1/45)	6.7% (2/30)	0.560
Late mortality	4.3% (4/93)	2.9% (1/34)	1	2.2% (1/45)	3.3% (1/30)	1
Reoperation	5.4% (5/93)	5.9% (2/34)	1	2.2% (1/45)	3.3% (1/30)	1

In our study, we found that high postoperative CVP is an independent risk factor for postoperative death. We divided the patients into three equal cohorts according to postoperative CVP and found that when postoperative CVP > 8 mmHg, early and overall mortality increased significantly. Previous studies have suggested that high CVP is independently related to all-cause mortality of patients undergoing cardiovascular cardiopulmonary bypass (19). Williams et al. (20) found that for every 5 mmHg increase in CVP at 6 h after admission to the ICU, the risk of early death and renal failure increased significantly, and this relationship was continuous and independent of cardiac index. We believe that high CVP in neonatal patients has a significant impact on the functioning of organs with dysplasia. Since venous return is determined by the difference between the mean systemic filling pressure and CVP, an increase in CVP will affect venous return; moreover, an excessive increase can even hinder venous return, leading to a rise in venous pressure and reduction in cardiac output. Furthermore, increasing venous pressure causes rising renal subcapsular pressure, thereby reducing renal blood flow and estimated glomerular filtration rate (eGFR). Previous studies have also confirmed that higher levels of CVP result in reduced eGFR, and that eGFR can affect survival by different mechanisms (21). On the other hand, the newborn heart is an immature myocardium, which has poor tolerance to high load. With an increase in CVP, the increased volume load causes end-diastolic volume to increase, which stretches myofibrils and impairs the inherent contractility of the myocardium, which in turn can lead to heart failure.

At the same time, elevated CVP may be associated with hypervelocity volume resuscitation, cardiac tamponade, valvular regurgitation, right ventricular dysfunction, and other potential ailments. This indicates that postoperative cardiac dysfunction or hemodynamic instability predicts poor prognosis and high mortality. We found that compared with the other patients, those with postoperative CVP > 8 mmHg were at higher risk of postoperative pulmonary arterial hypertension (PAH) (27.3% [24/88] vs. 41.0% [16/39], respectively), or moderate or above postoperative PAH (8.0% [7/88] vs. 15.4% [6/39], respectively). However, this difference did not reach statistical significance. For postoperative management of these patients, we usually choose to adjust the positive end expiratory pressure of the ventilator first, and then use related drugs (e.g., treprostinil) and a carbon monoxide machine followed by ECMO therapy.

### Postoperative PVO and Reoperation

In this study, the incidence of postoperative PVO and reoperation were 11.8% (15/127) and 5.5% (7/127), respectively. Postoperative PVO is mainly anastomotic stenosis and branch pulmonary vein stenosis. We found that preoperative acidosis and prolonged CPB time were risk factors for postoperative PVO. We believe that preoperative acidosis and longer CPB time may indicate those patients who are in poor preoperative condition and therefore more difficult to operate on. The venous tissue of neonates is more fragile and delicate. Clamping and fixing the pulmonary vein during repair is more likely to cause injury. These factors contribute to postoperative pulmonary vein intimal hyperplasia and anastomotic stenosis (22). At the same time, compared with anastomotic stenosis, stenosis of the branch pulmonary vein is more difficult to completely relieve, so prognosis for these patients was poor after reoperation in our study.

This study also confirmed that mixed TAPVC was more prone to postoperative PVO than other types (37.5 vs. 10.0%). In our study, mixed TAPVC was identified as an independent risk factor for postoperative PVO. Since there is no complete single pulmonary vein confluence, most patients with mixed TAPVC usually have multiple anastomoses. Bando et al. (5) found that the confluence of venules and diffuse pulmonary vein stenosis were risk factors for adverse results. Seale et al. (23) demonstrated that patients without a single anastomosis were at greater risk for postoperative PVO, and most mixed TAPVC cases fell into this category. As a result, the risk of postoperative restenosis in patients with mixed TAPVC is significantly higher (24, 25). Karamlou et al. (7) also suggested that patients with mixed TAPVC were more likely to need reoperation. Therefore, we should note that patients with mixed TAPVC and pulmonary vein dysplasia have a higher risk of postoperative PVO.

## Limitations

This study is subject to the common limitations of single-center and retrospective studies. Subgroups in the study cohort could not be completely randomized, and there may have been some statistical bias. In this study, we could not preclude results being influenced by differences between the surgical period and surgeon, which may limit meaningful comparison of outcomes between the sutureless and conventional techniques. For study of postoperative PVO and reoperation, a longer follow-up period is warranted. Patients' symptoms and families' financial condition contributed to decision-making for reoperation to address postoperative PVO, which also affected accuracy of the follow-up information.

## Conclusion

Although the mortality rate of neonatal TAPVC has dropped to 6.7% in the past 5 years, it is still relatively high. The risk factors for postoperative death are preoperative acidosis, prolonged CPB time and increased postoperative CVP. Patients with preoperative acidosis and mixed TAPVC are at higher risk of postoperative PVO. The mortality of neonates with an average CVP > 8 mmHg at 24 h after surgery was significantly higher than for other patients.

## Data Availability Statement

The raw data supporting the conclusions of this article will be made available by the authors, without undue reservation.

## Author Contributions

EJ, HQ, and XLiu wrote the manuscript and conducted statistical analysis. WX and TL conducted data inspection and validation. JZ and JCe provided funding support and supervision. JChe, SW, and XLi revised the manuscript. All authors contributed to the article and approved the submitted version.

## Funding

This work was supported by the Science and Technology Planning Project of Guangdong Province (2019B020230003; 2017B090904034; 2017B030314109; and 2018B090944002), Guangdong Peak Project (DFJH201802), and Guangdong Provincial Medical Science and Technology Research (A2020029).

## Conflict of Interest

The authors declare that the research was conducted in the absence of any commercial or financial relationships that could be construed as a potential conflict of interest.

## Publisher's Note

All claims expressed in this article are solely those of the authors and do not necessarily represent those of their affiliated organizations, or those of the publisher, the editors and the reviewers. Any product that may be evaluated in this article, or claim that may be made by its manufacturer, is not guaranteed or endorsed by the publisher.
